# Embedding mentoring to support trial processes and implementation fidelity in a randomised controlled trial of vocational rehabilitation for stroke survivors

**DOI:** 10.1186/s12874-021-01382-y

**Published:** 2021-10-03

**Authors:** Kristelle Craven, Jain Holmes, Katie Powers, Sara Clarke, Rachel L. Cripps, Rebecca Lindley, Julie Phillips, Ruth Tyerman, Christopher McKevitt, David Clarke, Kathryn Radford

**Affiliations:** 1 Rehabilitation & Ageing Research Group, Injury, Inflammation and Recovery Sciences, School of Medicine, University of Nottingham, Nottingham, NG7 2UH UK; 2 School of Population Health & Environmental Sciences, King’s College London, Addison House, London, SE1 1UL UK; 3 Academic Unit for Ageing and Stroke Research, Leeds Institute of Health Sciences, University of Leeds, Woodhouse Lane, Leeds, LS2 9UT UK

**Keywords:** Mentoring, Occupational therapy, Stroke, Vocational rehabilitation, Randomised controlled trial, Trial processes, Adherence, Implementation fidelity, Process evaluation

## Abstract

**Background:**

Little guidance exists regarding how best to upskill and support those delivering complex healthcare interventions to ensure robust trial outcomes and implementation fidelity. Mentoring was provided to occupational therapists (OTs) delivering a complex vocational rehabilitation (VR) intervention to stroke survivors. This study aimed to explore mentors’ roles in supporting OTs with intervention delivery and fidelity, and to describe factors affecting the mentoring process and intervention delivery.

**Methods:**

Quantitative data (duration, mode and total time of mentoring support) was extracted from mentoring records and emails between mentors and OTs, alongside qualitative data on barriers and facilitators to intervention delivery. Semi-structured interviews with mentors (*n* = 6) and OTs (*n* = 19) explored experiences and perceptions of intervention training, delivery and the mentoring process. Mean total and monthly time spent mentoring were calculated per trial site. Qualitative data were analysed thematically.

**Results:**

Forty-one OTs across 16 sites were mentored between March 2018 and April 2020. Most mentoring was provided by phone or Microsoft Teams (range: 88.6–100%), with the remainder via email and SMS (Short Message Service) text messages. Mentors suggested strategies to enhance trial recruitment, improved OTs’ understanding of- and adherence to trial processes, intervention delivery and fidelity, and facilitated independent problem-solving. Barriers to mentoring included OT non-attendance at mentoring sessions and mentors struggling to balance mentoring with clinical roles. Facilitators included support from the trial team and mentors having protected time for mentoring.

**Conclusions:**

Mentoring supported mentee OTs in various ways, but it remains unclear to what extent the OTS would have been able to deliver the intervention without mentoring support, or how this might have impacted fidelity. Successful implementation of mentoring alongside new complex interventions may increase the likelihood of intervention effectiveness being observed and sustained in real-life contexts. Further research is needed to investigate how mentors could be selected, upskilled, funded and mentoring provided to maximise impact. The clinical- and cost-effectiveness of mentoring as an implementation strategy and its impact on fidelity also requires testing in a future trial.

**Trial registration:**

ISRCTN, ISRCTN12464275. Registered on 13th March 2018.

**Supplementary Information:**

The online version contains supplementary material available at 10.1186/s12874-021-01382-y.

## Background

Trialists are responsible for ensuring that those delivering trial interventions are adequately prepared and supported to implement them with fidelity to ensure robust trial outcomes [1] and optimal participant outcomes [2]. While guidance describing best practice in trial management and the role of healthcare staff to deliver trial interventions exists [3], evidence on how best to upskill and support staff to deliver trial interventions as opposed to using experts to deliver it is lacking. This is particularly pertinent in the UK, where the funding infrastructure separates research and intervention costs and there is a requirement to use National Health Service (NHS) staff to deliver trial interventions. The premise is worthy, if staff can be trained to deliver trial interventions in real-life contexts, trial findings will be contextually relevant and may rapidly translate into practice. However, there are many obstacles which interfere with intervention delivery [4, 5] that need addressing to maximise trial outcomes.

Multiple strategies may be needed to support staff because barriers to delivering complex interventions with fidelity exist at organisational and practitioner levels [1, 6–8], although evidence on the effectiveness of these strategies is limited [9]. In a systematic review, barriers to fidelity in delivery of rehabilitation to people with long-term neurological conditions included lack of availability of training and therapists’ lack of confidence to deliver new interventions, especially where there was a gap between training and initiating intervention delivery [4]. Implementing new interventions in clinical practice or in a trial context may require changes to the staff member’s knowledge, skills, confidence, and attitudes [10, 11], and how they put new learning into practice is complex [9, 12–14]. The Cochrane Effective Practice and Organisation of Care (EPOC) Group categorised implementation strategies in a taxonomy [15, 16], including those targeting healthcare staff such as occupational therapists (OTs). Implementation strategies in stroke rehabilitation commonly included educational materials and meetings, outreach visits and support from opinion leaders [17], i.e., trustworthy individuals educating staff on ways to best apply evidence to inform their practice [18].

A novel approach to support intervention delivery was taken in the Facilitating Return to work through Early Specialist Health (FRESH) feasibility trial of vocational rehabilitation (VR) for traumatic brain injury survivors [19]. A training package comprising face-to-face teaching, an intervention manual and monthly mentoring for NHS OTs delivering the intervention was provided. Key components of mentoring included determining whether the OTs were delivering the intervention as intended and providing feedback to encourage implementation fidelity. One unintended benefit was that the mentoring led to early identification of trial-related issues, timely trouble-shooting and local problem-solving [5]. The FRESH mentoring approach was then adapted to provide clinical supervision and promote implementation fidelity among OTs in the UK-based RETurn to work After stroKE (RETAKE) trial [20].

The RETAKE trial aims to determine whether providing early stroke-specialist vocational rehabilitation plus usual NHS rehabilitation is more clinically and cost-effective for supporting post-stroke return to work than usual care (UC) alone [20]. This complex intervention aligns with current NHS outcomes, government policy and clinical guidelines for return to work after stroke [21–25], and is described elsewhere [26]. The RETAKE intervention is individually tailored, commencing within 12 weeks of stroke onset and delivered in participants’ homes or workplaces by NHS OTs for up to 12 months. In the RETAKE trial, two OTs were recruited to deliver the intervention per NHS site. This study was completed as part of the RETAKE trial process evaluation; a mixed-methods approach was taken to describe and explore the mentoring provided to RETAKE OTs.

## Methods

The aims were:

1. To explore mentors’ roles in supporting OTs with intervention delivery and implementation fidelity.
2. To describe factors affecting the mentoring process and intervention delivery.

The qualitative sections of this paper are reported in accordance with the Standards for Reporting Qualitative Research (SRQR) guideline [27]. The methods described henceforth were conducted in alignment with the principles of Good Clinical Practice (GCP) [28], and Declaration of Helsinki (1996) [29]. Further details of ethical approval and participant consent are included in the declarations section.

### Training and mentoring in the RETAKE trial

A description of the core elements of the RETAKE mentoring approach can be found in Additional file 1. Prior to intervention delivery, OTs attended a two-day educational meeting where they were provided with evidence on the trial intervention and taught how to deliver it and complete the trial paperwork. Educational materials were provided (e.g., intervention manual, letter and report templates). The OTs’ competency to deliver the RETAKE intervention was assessed via case vignettes immediately following training and 6 months post-randomisation of their first participant. Low competency scores indicated that additional training/mentoring support was required, such as email support or one to one phone calls alongside group mentoring. Following initial intervention training, monthly group mentoring sessions were provided for all OTs via teleconference or Microsoft Teams. Attendees at each session included a mentor and OTs across two trial sites. Following each session, mentors completed an electronic mentoring record (Additional file 2) recording date and duration of the session, OT attendance (including reasons for non-attendance), issues and actions relating to RETAKE OTs, clinical matters, implementation of the intervention, and trial process issues. OTs could contact their mentors via phone, text or email for ad-hoc support outside of sessions; mentors recorded ad-hoc conversations via mentoring records or emails. A one-day refresher training was held 6 months later to reinforce the intervention process, facilitate peer sharing of resources and problem-solve implementation fidelity issues.

Mentors were purposively approached based on national recognition as experts, with substantial experience delivering VR to stroke and/or acquired brain injury patients. All were members of the Royal College of Occupational Therapists (RCOT) Specialist Section in Work or RCOT Specialist Section for Neurological Practice. All but one had been involved in supporting implementation of VR interventions in previous trials for people with traumatic brain injury [5] and stroke [30]. They were approached by the Chief Investigator and their time costed into the grant application. Two mentors held PhDs; they delivered RETAKE intervention training and had prior experience of supporting VR delivery in research. They were embedded within the RETAKE research team and attended trial management group meetings. All mentors received training in the RETAKE mentoring process, potential sources of contamination between trial arms and how to reduce contamination risks, and how to use teleconferencing to deliver mentoring. The mentors’ demographic details are presented in Table 1.

**Table 1:** Demographic details of mentors and OTs interviewed

	Mentors	OTs
**Male**		1 (5.3)
**Female**	6 (100)	18 (94.7%)
**Years qualified as an OT**	Mean: 29.3 (range: 18–40)	Mean: 18.7 (range: 6–34)
**Years’ experience in:**		
Mentoring (or supervising)	Mean: 13.4 (range: 2–35)	
Stroke rehabilitation	Mean: 13.3 (range: 2.5–22)	Mean: 10.1 (range: 0–32.3)
Vocational Rehabilitation	Mean: 17.8 (range: 9.5–25)	Mean: 3.3 (range: 0–12)
Research	Mean: 6.5 (range: 0–14)	
**Previous research roles** (note: multiple roles were reported by some mentors)	Research Occupational Therapist (1) Mentor (3) Trainer for clinicians and researchers (2) Completed PhD (2) Research Assistant (2) Research Fellow (2)	
**Employment circumstances/clinical roles during RETAKE period** (note: multiple roles were reported per some individuals due to changes during RETAKE period)	Retired (1) Employed in NHS senior position (2) Seconded from NHS to UoN^a^ (1) Researcher at UoN (1) Private VR business owner (2) Retired/moved abroad later on in study (2)	Clinical Occupational Therapist (14) Occupational Therapy Team Leader (3) Independent Occupational Therapist (1) Senior Research Assistant (1)
**NHS band**		Band 6 (12) Band 7 (7)
**Education**^**b**^	DipCOT^c^ (1) Bachelor’s degree (1) Master’s degree relating to OT (1) Postgraduate certificate in VR (1) PhD in VR research (2)	DipCOT (2) Bachelor’s degree (16) Master’s degree relating to OT, healthcare, or research methods (4)

^a^eUoN = University of Nottingham; ^b^ Education data missing for 3 OTs; ^c^ DipCOT = Diploma of the College of Occupational Therapists

### Sampling and recruitment

All six mentors were invited to participate in interviews because they had first-hand experience of mentoring to support OTs’ and insight into real-world factors influencing the implementation of the RETAKE intervention. OTs were sampled in two ways, one OT from each site was purposively selected and invited to interview to ensure geographical spread. In addition, some OTs delivering the intervention to participants randomly selected as ‘longitudinal case studies’ were interviewed.

Prior to interviews, mentors and OTs were given study information and opportunity to ask questions. Mentors were geographically dispersed, so consent was obtained verbally at the time of interview. OTs consented in writing at the trial outset, to provide information for the trial duration.

### Data collection

#### Semi-structured interviews

All interviews were semi-structured and audio-recorded. Interviews with mentors were conducted by two research assistants (RAs) via telephone or face-to-face for up to 1 h in February 2020. The purpose was to explore mentors’ thoughts on the mentoring process, including how it may have influenced trial processes and intervention fidelity. One RA interviewed the OTs face-to-face between May 2019 and February 2020 for up to 1 h. Interviews took place once OTs had started seeing participants, to explore their experiences of training, mentoring and intervention delivery. Interview topic guides were informed by Normalisation Process Theory (see Additional files 3 and 4).

#### Documentary analysis

The number of sessions, emails and mentoring time provided to each OT during the study period was extracted from mentoring records and emails. Duration for individual emails was recorded as 10 min, unless otherwise specified. Emails between OTs and mentors were collated by researchers on an ongoing basis, and relevant, anonymised qualitative data extracted. Data generated between March 2018 and April 2020 enabled data capture across multiple sites from site opening until participant discharge from the RETAKE intervention (12-months post-randomisation). Examination of mentoring records and emails enabled description of factors influencing implementation fidelity and intervention delivery, and exploration of different dimensions of mentoring support during the RETAKE trial.

Data collection and analyses were underpinned by Normalisation Process Theory (NPT) [31]. NPT has been used extensively within implementation research [32], and applied in the RETAKE process evaluation to evaluate how the intervention was implemented, embedded, and sustained. The core constructs of NPT (see Table 2) were applied in the current study because mentors advised OTs on intervention delivery throughout the RETAKE trial, and were thus central to the implementation process. Similarly, OTs’ experiences of training and access to mentoring provided direct insights into the implementation process.

**Table 2:** Definitions of the core constructs of NPT, adapted from May et al., 2015 [31]

NPT core construct	Construct components
**Coherence:** sense-making, when faced with problems of operationalising practices	**Differentiation:** understanding how things are different **Communal specification:** people working together to have shared understanding of aims/objectives and expected benefits **Individual specification:** individuals understanding their specific tasks and responsibilities **Internalisation:** understanding the value, benefits, and importance of a set of practices
**Cognitive participation:** relational work to build and sustain community of practice	**Initiation:** whether or not key people are working to drive new/modified practices forward **Enrolment:** organising people to collectively contribute to work involved in new practices **Legitimation:** ensuring other participants believe they should be involved and can contribute **Activation:** collectively defining actions/procedures needed to sustain practice and stay involved
**Collective action:** operational work to enact practices	**Interactional workability:** how people interact with each other, artefacts, and other practices to operationalise in everyday settings **Relational integration:** knowledge work people do to build accountability and maintain confidence in a set of practices and each other as they use them **Skill set workability:** allocating work to people with the correct skills sets **Contextual integration:** allocating resources and executing protocols, policies and procedures (to manage set of practices)
**Reflexive monitoring:** understanding how the set of practices affect them and others	**Systemization:** collecting info to see how effective/useful a set of practices are (e.g., randomised controlled trials, anecdotes) **Communal appraisal:** people working together to evaluate the worth of a set of practices **Individual appraisal:** Individuals appraise how a set of practices affects them (as an individual) and contexts in which they are set **Reconfiguration:** appraisal work leading to attempts to redefine procedures or modify practices

### Data analysis

Total mentoring time per OT and per site were summed from March 2018 until April 2020 (Table 3). Time spent per mode (e.g., phone/Microsoft Teams) was calculated, as well as monthly averages per site.

**Table 3:** Breakdown of mentoring support provided to OTs at RETAKE sites: March 2018–April 2020

Site	Total number of months in study (as of 1st April 2020)	Total time mentoring provided per site via phone/Microsoft Teams and email (minutes) ^**a**^	Percentage of mentoring provided per site via phone or Microsoft Teams	Number of ESSVR participants per site between opening and April 2020 ^**b**^	Number of OTs delivering intervention per site during study period ^**c**^	Average amount of mentoring per site per month (minutes)	Average number of ESSVR participants per OT
1	25	2815	94.0	26	3	113	9
2	25	1086	99.1	17	2	43	9
3	24	2514	91.6	21	2	105	11
4	24	2324	93.5	19	4	97	5
5	24	2840	92.3	21	3	118	7
6	24	2160	97.2	30	3	90	10
7	24	2906	96.9	20	2	121	10
8	23	2390	95.0	8	3	104	3
9	22	1119	100.0	22	2	51	11
10	22	1206	95.9	14	2	55	7
11	22	1605	97.5	14	3	73	5
12	18	1600	94.4	13	2	89	7
13	18	1053	97.2	13	2	59	7
14	17	1400	96.4	6	2	82	3
15	8	612	100.0	7	4	77	2
16	14	1490	92.6	11	2	106	6

^a^It was anticipated that pre-planned mentoring sessions would last approximately 60 min, with two sites attending each session. The grand total for this column does not thus reflect the total time mentoring was provided across sites

^b^Each ESSVR participant was treated for up to 12 months post-randomisation

^c^1–2 OTs delivered the RETAKE intervention per site at any one time; OTs who left were replaced

Qualitative data from mentoring records, emails, and interviews with mentors and OTs were analysed in three separate thematic analyses [33] using Nvivo™ version 12 [34], adopting a staged approach to ensure trustworthiness. This approach, described in more detail below, was designed to ensure a consistent approach to analysis of each data set. Stages included independent coding, development of coding frameworks and thematic narrative summaries and then synthesis across the qualitative data sets. An inductive approach ensured coding and theme development were data-led. A subsequent deductive approach involved reviewing data against the research questions, NPT constructs and components, and intervention logic model. This combined approach was adopted to direct focus to the research questions, whilst allowing for any important, unexpected issues to be identified.

#### Analyses of interviews with mentors and OTs

Two RAs independently coded two mentor interview transcripts identifying themes and sub-themes; the same approach was taken with four OT interview transcripts. RAs then compared coding and agreed a provisional coding framework to be applied to the remaining interview transcripts. During analysis new nodes were added if indicated. One RA developed summary memos synthesising findings per theme using the deductive approach described above. Memos were reviewed by the team to clarify and confirm explanations.

#### Analysis of mentoring records and emails

Two RAs coded data extracted from the mentoring records and emails to identify provisional themes and sub-themes, and then met to review coding and agree themes and sub-themes. Themes and corresponding data were checked independently by a third RA, and disagreements resolved through discussion. Themes and sub-themes were then reviewed by the team.

### Researcher characteristics and trustworthiness

RAs were Psychology graduates with qualitative research experience. To increase rigour, various measures were undertaken [35, 36]. To ensure credibility, annotations were made during coding and theme collation stages in analyses, recording RAs’ thoughts on patterns emerging through the data. RAs and senior researchers regularly discussed development of interpretations and plans for action. An RA examined findings across the qualitative and quantitative analyses and across trial sites as part of data triangulation [35] to address the following research questions: 1) How was mentoring delivered?; 2) How did mentors support OTs with intervention delivery?; and 3) What have we learned? Transferability was ensured through description of study boundaries, including: Number of sites contributing to the data; numbers of mentor and OT participants involved; and data collection methods. Confirmability was ensured through multiple RAs and senior research staff being involved in creation of the interview topic guides and in review of themes in qualitative analyses.

## Results

Table 1 provides demographic details for mentors (*n* = 6) and OTs (*n* = 19) interviewed. Data were extracted from 184 mentoring records and 240 emails between March 2018 and April 2020.

Themes identified across the qualitative analyses related to delivery of mentoring, benefits of mentoring for OTs and for trial implementation, facilitators and barriers to mentoring, and implications for future provision of similar mentoring approaches. Themes, sub-themes and examples of data are presented as follows. Within the text and Table 4 NPT constructs and components have been highlighted, and influences on the mentoring process and RETAKE intervention delivery identified to help explain findings. Within the ‘delivery of the mentoring’ theme,’ reference has also been made to quantitative data from the mentoring records to demonstrate the mode and time spent mentoring each site per month.

**Table 4:** NPT constructs and components coded per sub-theme

Themes and sub-themes	NPT constructs and components	Facilitators to mentoring and RETAKE intervention delivery, coded by NPT components (source of data)	Barriers to RETAKE intervention delivery, coded by NPT components (source of data)
**Delivery of the mentoring**			
Preparation of mentors to deliver the mentoring	Coherence: - *Communal specification* Cognitive participation: *- Initiation*	*Communal specification*: RETAKE training increased mentors’ understanding of the RETAKE intervention, ensuring they had a shared understanding of its aims/objectives and expected benefits (Mentor interviews) *Initiation:* Attendance of RETAKE training increased mentors’ understanding of the clinical/research processes and OT’s unique contextual challenges. This, along with their VR backgrounds, suggests they were the right people to drive delivery of the RETAKE intervention (Mentor interviews)	
Monthly mentoring sessions and additional support	Collective action: *- Interactional workability*	*Interactional workability:* The adaptability of the mentors (e.g., devising strategies) and cooperation from and between OTs suggests they interacted well with one another to operationalise the group teleconference format (Mentor interviews)	
**The benefits of mentoring for OTs and trial implementation**			
Strategies to facilitate recruitment into the RETAKE trial	Coherence: - *Internalisation* Cognitive participation: *- Activation* *Collective action:* *- Interactional workability*	*Activation:* Mentors defined actions with OTs to prompt recruiters and help on wards, thus helping to sustain increase recruitment rates (Mentoring records/emails) *Interactional workability:* Following communication with mentors, OTS and Principal Investigators worked together to prompt recruiters and help on wards, thus helping to increase and sustain recruitment (Mentoring records/emails)	*Internalisation:* Lack of referrals occurred in one site due to staff not understanding the potential value of the RETAKE intervention for stroke survivors with low level impairments (Mentor interviews)
Support with trial processes	Coherence: *- Individual specification* Cognitive participation: *- Activation* Collective action: *- Relational integration*	*Individual specification:* Mentors helped OTs understand their tasks/responsibilities in trial processes, e.g., trial paperwork (Mentor interviews) *Activation:* OTs did not always have time to complete RETAKE paperwork. Mentors provided practical solutions to speed up and sustain paperwork completion (Mentoring records/emails) *Relational integration:* Regular communication across the central RETAKE team, mentors, and OTs helped build accountability and confidence in each other’s abilities (e.g., OTs avoiding contamination) (Mentor interviews)	
Support with applying newly acquired knowledge	Coherence: *- Individual specification* Collective action: *- Contextual integration*	*Individual specification:* Mentors helped OTs to understand their specific tasks and responsibilities when creating an individually tailored VR treatment plan (Mentoring records/emails) *Contextual integration:* Mentors signposted OTs to relevant local and national resources to support their delivery of the RETAKE intervention (Mentoring records/emails)	
Support for delivering the intervention with fidelity	Coherence: *- Internalisation* *- Differentiation* *- Individual specification* Cognitive participation: *- Initiation* *- Enrolment* *- Legitimation* *- Activation* Collective action: *- Interactional workability*	*Internalisation:* All OTs interviewed believed in the value of all or some the RETAKE intervention’s core components (OT interviews) *Individual specification:* Mentors helped OTs understand their tasks/responsibilities for delivering the intervention with fidelity, e.g., to start working with participants within 12 weeks of their stroke, to avoid discharging participants as soon as they had returned to work (Mentor interviews, mentoring records/emails) *Legitimation:* Mentors reassured OTs that they were “on the right track” and had not failed if a participant had not been able to return to work (Mentor interviews) *Legitimation:* OTs felt able to contact mentors if they needed reassurance they had taken the right steps with RETAKE intervention delivery (OT interviews) *Activation:* Mentors supported OTs with knowing how to manage interactions with employers, including those who were difficult to engage (Mentor interviews) *Interactional workability:* Mentors supported OTs with knowing how to interact with their managers to operationalise RETAKE intervention delivery in their setting (Mentor interviews)	*Internalisation and differentiation:* Unsupportive managers may not have understood the potential value of the RETAKE intervention, nor understood how it differed from usual care (OT interviews, mentoring records/emails, mentor interviews) *Initiation and enrolment:* OTs were sometimes expected to deliver RETAKE in usual hours and were pressurised to stay on top of usual caseloads. Collective contribution to RETAKE intervention delivery may not have always been organised beforehand, and/or key senior staff involved to drive implementation of the intervention (Mentor interviews and mentoring records/emails) *Legitimation:* Some unsupportive managers may not have believed their OTs should be involved in RETAKE, e.g., some managers themselves were new had not been formally introduced to RETAKE (OT interviews)
Facilitation of independent problem-solving and peer support	Cognitive participation: *- Activation* Collective action: *- Interactional workability* *- Relational integration*	*Activation:* OTs’ learning was supported by mentors’ facilitation of conversations during mentoring sessions, enabling collective problem-solving to take place (OT interviews) *Interactional workability:* Some OTs had regular contact with site partners (e.g., peer supervision, joint visits to participants) to support operationalisation of RETAKE intervention delivery in their area (OT interviews) *Relational integration:* OTs had regular contact with mentors, other OTs, and site partners to freely discuss caseloads, thus building accountability and confidence in their RETAKE intervention delivery (OT interviews)	

### Delivery of the mentoring

#### Coherence and cognitive participation: preparation of mentors to deliver the mentoring

In addition to training on the RETAKE mentoring process, mentors reported that attending training for OTs at trial outset improved their understanding of trial processes and the RETAKE intervention. This ensured coherence by facilitating shared understanding of the intervention’s aims, objectives and potential benefits. Attending the training also enabled mentors to meet OTs face-to-face and learn about their backgrounds and possible site-related challenges.

*“It’s just nice to meet people and find out where they came from and also what the challenges were of working within their Trust.” [Interview with Mentor 1]*

All six mentors had extensive experience delivering VR, often with focus on neurological conditions such as stroke or traumatic brain injury. This experience enabled reflection on times they had been in similar situations and use of those experiences to guide OTs.

*“And obviously I’ve got experience to draw on as well so that if people hadn’t, you know, I can say, “Well, I had something similar and I found that this worked.” [Interview with Mentor 1]*

The combination of mentors’ VR backgrounds and understanding of the trial, RETAKE intervention and OTs’ potential site-related challenges exemplifies cognitive participation because it suggests they were the right people to support delivery of the RETAKE intervention.

#### Collective action: monthly mentoring sessions and additional support

Two mentors experienced initial difficulties knowing which OTs were speaking when delivering mentoring by teleconference. They developed strategies for overcoming this, such as asking OTs to say each other’s names when speaking, making notes per OT, and planning an agenda.

*“ … I’d never done that [teleconference mentoring]. So it was a little daunting for me and I had to sort of devise my own systems of how I recorded it and how it could be – how to recognise people’s voices … ” [Interview with Mentor 2]*

The adaptability of mentors and OTs in devising strategies to overcome initial challenges demonstrates collective action, through their work to operationalise the group teleconference format.

Mentoring records and emails demonstrated 41 OTs across 16 trial sites participated in mentoring (Table 3). It was mostly provided by phone or Microsoft Teams (range across sites: 88.6 to 100%), with the remainder via email. Where reported, reasons for non-attendance included OT sickness, annual leave, clinical commitments, technical issues, or redeployment during the Covid-19 pandemic. During this period, 9 OTs across 7 sites left RETAKE to go on maternity leave (*n* = 2), start new jobs in other Trusts or services (*n* = 6), or left due to pressures from their usual clinical role (*n* = 1). As Table 3 indicates, site 2 received the least mentoring with OTs receiving, on average, 43 min per month during the study period. In this site one OT left in August 2019 and was never replaced. The remaining OT was a manager and reportedly didn’t have time to attend mentoring sessions. Three sites received close to the expected 60 min of mentoring per month (sites 9, 10, and 13), and twelve sites received more time (range of averages per month: 73–121 min), which included additional mentoring via phone/Microsoft Teams and email. Known reasons for additional support included larger caseloads (so more participants to be discussed) in sites 1 and 6, OTs being less confident about delivering the intervention (sites 3, 7, 8 and 15), low scores on RETAKE competency assessments (sites 1, 4, 5, 7, 11, 15, and 16), and site-specific issues related to set-up, funding, intervention delivery, and management issues (sites 7, 11, 12, and 14). OT turnover at 7 sites (sites 1, 4, 5, 6, 8, 11, and 15) meant that replacement OTs required more support because they were new to the trial and intervention delivery.

Qualitative analyses of interviews and mentoring records and emails demonstrated that ad-hoc support was provided to OTs via phone, email and/or SMS text message. In contrast to data in Table 4, two mentors interviewed described receiving infrequent queries for additional support, although another reported that some OTs habitually phoned her for advice. This might be partially explained by the fact that mentors arranged additional mentoring sessions for OTs unable to make the planned session, thus increasing the total amount of support provided. Additionally, two mentors left during the study period, due to long-term sickness or moving overseas.

Six of the 19 OTs reported that monthly mentoring sessions plus ad-hoc, additional support provided enough opportunity to engage with their mentor and other OTs.

*“I think once a month is enough … But also, [mentor] has said that we can contact her in between. So if we've got something pressing, we can email her or have a conversation with her in between..” [OT 18 from site 10]*

### The benefits of mentoring for OTs and trial implementation

#### Coherence, cognitive participation and collective action: strategies to facilitate recruitment to the RETAKE trial

Delays in referrals prevented OTs from delivering early intervention to participants. One mentor reported that recruiters in one site lacked understanding as to why stroke survivors with low level impairments needed support for returning to work, demonstrating lack of understanding of the aims of the trial and intervention (i.e., coherence) among recruiters.

*“And they [recruiters] never gave a thought to what happens about work because there was always another service that would deal with that in their minds. So asking them to think differently was quite a challenge.” [Interview with Mentor 2]*

Mentoring records and emails indicated that recruitment was sometimes paused because recruiting staff did not have capacity to recruit new participants. In these instances, cognitive participation and collective action were demonstrated because mentors worked with OTs to define strategies to increase and sustain trial recruitment, e.g., OTs prompting recruiters to increase efforts and offering help in recruiting. Following this communication and by involvement of Principal Investigators (PIs), recruitment increased. Other solutions included clarification of eligibility queries and encouraging OTs to obtain consent to follow-up even if in doubt of eligibility.

#### Coherence: and collective action: support with trial processes

Across mentoring records and mentor interviews a key aspect of the mentors’ role at the trial outset included answering OT’s queries about trial processes, e.g., when and how to complete trial case reporting forms, writing letters to participants and employers. If mentors were uncertain they sought clarification from the trial team. One mentor described reassuring community-based OTs that they could visit participants in acute settings, ensuring participants were seen within trial timelines (initial assessments were due within 2 weeks of randomisation).

*“I was like well, you know, it’s been agreed as part of the trial that you can in-reach and you can go and see people in A&E, and it just took a lot of sort of reassuring them (laughs) that they weren’t breaking any rules … .” [Interview with Mentor 2]*

Mentors supported OTs in developing coherence by helping them understand their tasks and responsibilities in relation to the trial. According to mentors, the sessions provided a safe place to confidentially discuss caseloads. Mentoring records demonstrated that contamination issues (e.g., usual care therapists learning about and applying the RETAKE intervention) were discussed in sessions and actions planned. For example, seeking advice from the trial team, or advising the OT on direct action.

*“We discussed how to avoid contamination and agreed; see the pt alone at the end of the session for voc [vocational rehabilitation] conversation. Educate the other therapists not to copy what has been said/done by the RETAKE OT and just note pt seen by RETAKE OT in standard notes.” [Email from mentor to mentee OT, 14/01/19]*

Regular communication between OTs, mentors, and the trial team led to collective action through building of accountability and confidence in each other’s abilities (e.g., OTs avoiding contamination).

Mentoring records/emails also showed that OTs reported they did not always have enough time to complete trial documentation. Cognitive participation was demonstrated when mentors worked with OTs to collectively define practical solutions to speed up and sustain trial documentation .

*“[RETAKE OTs] said they were writing letters after seeing pts but also writing continuation sheets... We discussed this and they agreed they could write ‘see letter’ on the continuation sheet … ” [Mentoring Record 21/11/18]*

#### Coherence and collective action: support with applying newly acquired knowledge

Four mentors stated that many OTs needed their support to apply newly acquired knowledge of VR, and both mentors and mentoring records indicated that OTs were sometimes unsure what VR treatment plans would look like. Mentors supported OTs in developing coherence by them on the tasks and responsibilities required when creating VR treatment plans.

*“Therapist unclear what voc [vocational] support to offer at this point. Reassured ok still very early stages. Talked through establishing job demands, using job description/break down of job, relating to current activity, how to establish grade plan of activity at home to test out and build up skills.” [Mentoring Record 06/08/18]*

Mentors had extensive knowledge of resources to facilitate return to work after stroke. Mentoring records and emails indicated that many resources were provided by mentors to individual OTs, or to all OTs if seen as relevant to all. Collective action was evident with mentors advising OTs on use of these resources and signposting to relevant training to support their use.

#### Coherence, cognitive participation and collective action: support for delivering the intervention with fidelity

All 19 OTs interviewed demonstrated evidence of coherence, because they believed in the value of the RETAKE intervention. Their most frequently valued components including early intervention, case coordination and opportunity to build relationships between employers and participants. Ten OTs explained that mentoring facilitated intervention delivery through the advice and best practice examples shared. Interviews with mentors and mentoring records and emails indicated that mentors supported OTs in developing coherence by advising OTs on their tasks and responsibilities for delivering core components of the RETAKE intervention. Mentors also facilitated OTs’ adherence to the trial protocol during intervention delivery. For example, by advising OTs to start working with participants early (i.e., within 12 weeks of their stroke) and ensuring OTs did not discharge participants as soon as they had returned to work.

*“One participant discharged after 2 weeks back at work, had spoken with manager who was happy with performance. Advised that I felt this was too early for discharge and that she [OT] should be establishing a mechanism for keeping in touch and follow up.” [Mentoring Record 20/11/18]*

Mentors also provided guidance on participants’ access to benefits, tailoring interventions according to clinical assessment results, and working with employers.

*“I think one of the common themes that comes up is around talking about how they [OTs] manage employers, or how they manage the interactions with employers, or employers who are not really engaging with them.” [Interview with Mentor 5]*

OTs frequently lacked confidence in working with employers as it was unchartered territory for many. In addition to managing difficult or non-engaging employers, mentors supported OTs’ progression to cognitive participation by advising them on employer expectations, ensuring clarity in the structure, content and expected outcomes of interactions, and tailoring their communication styles. Four mentors explained that they provided reassurance to OTs, that what they were doing- or had done was appropriate or, “on the right track.” Mentors demonstrated cognitive participation by supporting OTs in believing they were contributing to the RETAKE trial. Further evidence for this came from OTs reporting occasional checks with mentors that they were following the right steps with intervention delivery, and from mentoring records and emails.

*“You wanted to just to check you had thought of everything – which you had. Well done.” [Email from mentor to mentee OT, 05/02/2020]*

A common issue was that OTs were hindered from starting the RETAKE intervention early, and delivering it at the required intensity, duration, and dose. Across mentor interviews and mentoring records and emails, common reasons included staff shortages and pressures from UC workloads. Three mentors and several mentoring records and emails highlighted unsupportive management issues, leading to increased pressures on OTs to prioritise UC over RETAKE, refusal to let OTs take on any more RETAKE participants, and pressure to drop out of RETAKE altogether. In these instances, coherence and cognitive participation did not seem evident among managers; mentors responded by engaging in collective action to support OTs.

*“[RETAKE OT] had been told by their manager that their usual care caseloads takes priority as that was what they were commissioned to do but you rightly reminded them that the trust was being paid to carry out research so the RETAKE pts must be seen as prescribed.” [Email from mentor to mentee OT, 13/02/19]*

OTs across two sites reported that where their managers had not been supportive it had been due to a change in management during the RETAKE study period, and the new managers not having had a formal introduction to RETAKE.

“ *… with my new manager who didn’t know anything about Retake, she kind of inherited our team, I had to explain it all to her and I am not sure, because she has just got so much to do, they have restructured and she has got loads of teams now … I am not sure if she ever really kind of understood what was going on with it … ” [RETAKE OT 30, Site 15]*

#### Cognitive participation and collective action: facilitation of independent problem-solving and peer support

Cognitive participation was evident because five mentors reported encouraging OTs to independently problem-solve, with mentors providing guidance where necessary. All OTs in the session were encouraged to share advice to facilitate peer support.

*“I guess my role is to try and facilitate a discussion amongst the team where they can do some shared learning … it’s not about me giving answers but trying to facilitate a discussion to draw people in … ” [Interview with Mentor 3]*

OTs reported their learning was supported by this facilitation of meaningful conversations; seven agreed that mentoring sessions enabled problem-solving, through input from their peers and reassurance from the mentor.

*“ … if situations come up there are other people that can sometimes also give advice, you know, I tried this with my patient, or you can advise them on this or whatever...” [OT 36, Site 9]*

Frequent liaison with other RETAKE OTs at the same site (i.e., a site partner) was beneficial. Mentoring records showed that OTs within sites (and sometimes across sites) met regularly for peer support and visited participants together when an OT lacked confidence. OTs across 7 of the 11 sites represented in the interviews reported they were able to regularly gain peer support from site partners outside of mentoring sessions. Three of the 19 OTs interviewed reported that regular contact with their site partner provided a safety net and opportunity to talk freely about participants.

*“I work really closely with my other counterpart that’s doing the RETAKE. Having somebody else has been really, really valuable because you can’t talk to anyone at work about it. You don’t want to bug people sort of from RETAKE all the time.” [OT 6, Site 3]*

In contrast, four OTs with little contact with site partners reported feeling isolated.

*“I still feel quite alone in it all, I know [site partner] is there if need be, having said that we have been trying to communicate all day yesterday and failed.” [OT 24, Site 12]*

Overall, regular communication between mentors and OTs (including other OTs at the same site or different sites) enabled collective action because it provided opportunity to discuss caseloads and conduct joint visits, thus building accountability and confidence in intervention delivery and supporting local operationalisation of RETAKE intervention delivery.

### Facilitators and barriers to the mentoring process

#### Facilitators to the mentoring process

For mentors, the most important facilitator to the mentoring process was support from the RETAKE trial team, including two mentors who were also members of this team. Four mentors reported receiving support with administration, trial processes and/or mentoring itself (e.g., advice on communication style with mentees). Two mentors also mentioned time availability as a facilitator to the mentoring process, as being retired or working part-time enabled them to provide support when necessary.

*“I’ve always tried to keep the mentoring on a Monday because that’s the day I didn’t work … I certainly didn’t find it difficult if OTs contacted me to say, could we have a phone call?” [Interview with Mentor 5]*

#### Barriers to the mentoring process

In contrast, two mentors found it difficult to juggle mentoring with their usual work, leaving them “overwhelmed” as one mentor put it. Another reported having to conduct some mentoring sessions via phone in her car between home visits, and she found it difficult disengaging from one work activity to the next.

*“Monday was the only day that worked for all the people in each group as it were … the days where it was a challenge was where I had [home] visits … I would be in the car and I did one or two mentoring sessions on my phone in the car, just park up somewhere, and then went off to my next visit.” [Interview with Mentor 6]*

A common barrier to the mentoring process was non-attendance of OTs at mentoring sessions; this was reported by three mentors as well as mentoring records and emails. Mentors’ perceived reasons for non-attendance included OTs having competing priorities in their usual caseloads, OTs needing to cover for colleagues on maternity- or sick leave, a rigid ‘clocking off’ mentality preventing OTs from attending sessions outside of their working hours, and a lack of confidence in participating in sessions and/or delivering the intervention. One mentor put in additional effort to encourage an OT to attend mentoring, as evidence from her competency assessment suggested she might not fully understand her RETAKE OT role.

*“I’ve got a new person who hasn’t come into mentoring so I’ve just emailed them now to say, “Can we have a ten minute conversation?” … She thinks she knows what she’s doing but I’ve just marked her paper [written competency assessment] and she doesn’t … ” [Interview with Mentor 4]*

### Implications for future provision of mentoring

#### Suggestions for improving mentoring

Suggestions for improving mentoring related to how it was organised, delivered, and evaluated. The most common suggestion from mentors was that it should occasionally occur face-to-face, because it was easier to detect more when communicating this way. Other suggestions included arranging mentoring by site, mentors receiving feedback from OTs, formal evaluation of OTs’ letter-writing, and meetings between mentors to share experiences and discuss OTs’ training needs.

*“whether we look at training needs because … as they [OTs] get into the process of the RETAKE, that’s when they’re recognising the skills lack in certain areas and whether we can offer any form of training” [Interview with Mentor 5]*

Two of the 19 OTs felt there was insufficient time to discuss caseloads in a 60-min mentoring session; and felt the time could have been structured more efficiently. Four OTs felt mentoring would work better in group format, whilst three others felt it would work better as one-to-one sessions between mentors and OTs.

#### Future provision of mentoring alongside complex intervention delivery

Among OTs, mentoring was considered essential in supporting complex intervention implementation in a trial context. Fourteen of the 19 OTs interviewed stated that a mentoring system would be needed alongside future roll-out of a similar VR intervention.

*“But for me, if a trial is introducing a new way of working with therapists that involves a complex intervention, I would say it’s extremely valuable to have a mentoring system in place.” [OT 5, Site 3]*

One mentor expressed concern that if mentoring was not rolled out alongside similar complex interventions in NHS settings, the pressures of new referrals would result in the intervention becoming “watered down.” She stated that adequate mentoring support would be needed to ensure those delivering the intervention did not withdraw too soon and were equipped to handle novel situations and challenges. Half of the mentors explained that how mentoring would be set up and paid for, and who would be suitable to provide mentoring within the contexts of local organisations were key for consideration in future trials.

*“the likelihood is that there are going to be local issues that have to be solved. So, either somebody externally has to come in and champion those and try and sort those out, or you have to have internal champions who are willing to think about those implementation issues and look at various different ways that they can be solved from within the organisation.” [Interview with Mentor 1]*

Due to the large number of OTs potentially delivering the intervention at any given time, four OTs suggested a future regional hierarchy based on skillset, whereby the more experienced OTs train and support the less experienced OTs. Four OTs also suggested they themselves could act in a mentoring/supervisory role.

## Discussion

Little guidance exists regarding how best to upskill and support those delivering complex interventions to ensure implementation fidelity and robust trial outcomes. In the UK, adequate trial-focused training and support for NHS staff may mean trial findings are more contextually relevant and translate more easily into real-world clinical practice. This study examined mentoring provided to OTs delivering a complex VR intervention to stroke survivors in NHS settings. The aims were to explore mentors’ roles in supporting intervention delivery and implementation fidelity; and to explore factors affecting the mentoring process and intervention delivery.

### Mentors roles

Mentors’ roles included supporting OTs with coherence, cognitive participation, and collective action through advising them on adherence to core components of the RETAKE intervention, such as commencing intervention early, individual tailoring, communicating with stakeholders, and avoiding discharge of participants immediately following return to work (typically the endpoint of usual NHS rehabilitation). Over half of OTs interviewed felt that mentoring facilitated their intervention delivery through provision of advice and best practice examples, suggesting that they valued mentors’ expertise and saw them as opinion leaders in VR. Similarly, in a Cochrane review [18] health professionals’ compliance with evidence-based practice improved on average by 10.8% when they received input from opinion leaders. Mentors also engaged in cognitive participation by facilitating independent problem-solving among OTs when issues arose, which is important because it encourages new thinking styles, behaviours, and independence among mentees [37]. Both OTs and mentors saw value in mentoring being provided alongside future complex intervention delivery to ensure fidelity, providing further support for use of opinion leaders [4, 38, 39]; and these findings may be replicated in future trials. RETAKE mentoring aligns with the UK government’s vision for clinical health research [40], which includes empowering staff to contribute to NHS clinical research and innovative research designs incorporating virtual processes and technologies (e.g., RETAKE mentoring was delivered via teleconference or Microsoft Teams).

Mentors also supported OTs with coherence and cognitive participation, through advising on trial processes relating to trial documentation completion, contamination, and recruitment. Support with trial documentation completion appears a novel finding in research, noteworthy because complicated trial documentation has been cited a key barrier to clinicians participating in practice-based research [41, 42]. Incomplete or missing trial documentation could have influenced accuracy of findings from RETAKE’s embedded economic and process evaluations. Mentors additionally engaged in collective action by alerting the trial team to potential contamination issues as they arose, reducing risk of minimisation of difference between trial arms [43]. Recruitment in sites was unofficially paused at times due to limited capacity of recruiting staff and was difficult to manage from the trial management perspective. Mentors helped by engaging in cognitive participation, i.e., through defining strategies with OTs to increase and sustain recruitment. Similarly, in a VR study using an identical mentoring model to RETAKE [5], mentoring gave timely insight into trial-related issues, enabled quick responses to support intervention delivery and facilitated implementation fidelity within OTs’ local contexts.

### Factors affecting the mentoring process and intervention delivery

Barriers to delivering the RETAKE intervention with fidelity included heavy usual caseloads and staff shortages. Similar issues were reported as barriers to nurses’ delivery of a family violence screening and care model [44]. The authors linked these barriers to key people not being involved to motivate and organise people and collectively define actions needed to sustain intervention delivery (i.e., lack of cognitive participation). Similarly in RETAKE, unsupportive managers pressurised OTs to prioritise UC over RETAKE or even drop out of RETAKE altogether, demonstrating lack of cognitive participation and collective action. These managers may not have developed coherence and lacked understanding of the potential benefits and differences of the RETAKE intervention compared with UC, possibly due to limited research experience. In future trials, trial-related managerial issues should be highlighted to the trial team, so that NHS managers can be educated on research processes and actions needed to sustain local intervention delivery. Importantly, the above findings suggest that RETAKE mentors took on roles akin to PIs and service managers, engaging in cognitive participation and collective action early on, e.g., to support trial processes and advise on interactions with managers to operationalise local RETAKE intervention delivery.

RETAKE mentors were funded through research budgets, but to ensure successful future roll-out alternative methods of resourcing this support would require consideration. Some OTs suggested a regional hierarchy based on skillset, whereby more experienced OTs train and support less experienced OTs. If the experienced OTs were considered opinion leaders their input could facilitate cost-effective intervention delivery, where funding for mentoring could be offset by savings through effective implementation. Such an approach would require justification and testing through implementation research [45].

Another point to consider is how future suitable mentors would be selected and upskilled. RETAKE mentors received training on the mentoring process and benefitted from attending RETAKE intervention training to improve their understanding of training, trial processes, the intervention, and OTs’ backgrounds. It is uncertain how this training would be funded and delivered within or across local contexts. The Royal College of Occupational Therapists (RCOT) trained health and work champions (i.e., opinion leaders) to deliver training to other staff within local organisations, enabling them to deliver brief VR advice to patients wishing to return to work [46]. It is possible professional bodies could train future mentors using similar methods. However, it remains unclear which mentoring model would be most effective alongside wide-scale roll-out of RETAKE or similar complex interventions. In RETAKE mentors with concurrent clinical roles struggled to balance mentoring and clinical workloads. Others with protected time for mentoring experienced no problems, which is an important consideration for future research.

### Strengths and limitations

This research had multiple strengths. Firstly, the thematic analysis of qualitative data in this study followed a systematic multi-step approach and included standard measures in order to increase rigour and trustworthiness.

The use of NPT as a sensitising framework for interpreting findings is considered a strength, as it offers insight into the mechanisms that may affect implementation of this intervention in the future.

We acknowledge that the sample of mentoring records and emails which were analysed covered a set time period which, although large, did not include data from later stages of the trial such as issues around closing sites or impact of the COVID-19 pandemic. The sample size of 6 mentors and 19 OTs can be considered small in relation to the size of the RETAKE trial. However, it included all of the mentors and at least one OT from each site and from different time points in the intervention delivery process. These recruitment methods, using purposive sample, together with the methods of analysis and use of NPT suggest that this sample generated data which are likely to be transferable to similar studies and settings [47].

## Conclusion

Little guidance exists on how best to support those delivering complex interventions to ensure robust trial outcomes and implementation fidelity. Study findings indicated that RETAKE mentoring enhanced trial recruitment, improved OTs’ understanding of- and adherence to trial processes, supported implementation fidelity and intervention delivery, and aided OTs’ development of problem-solving skills. It remains unclear to what extent RETAKE OTS would have been able to deliver the intervention without mentoring support or how this might have impacted implementation fidelity. Successful implementation of mentoring alongside such interventions may increase the likelihood of intervention effectiveness being observed and sustained in real-life contexts. Further research is needed to investigate the benefits of this approach for other complex interventions, including how mentors could be selected, upskilled, funded and mentoring provided to maximise impact. To ensure those implementing new complex interventions into clinical settings invest in the whole package, the clinical- and cost-effectiveness of mentoring as an implementation strategy and its impact on fidelity require testing in a future trial.

## Supplementary Information

**Additional file 1.**

**Additional file 2.**

**Additional file 3.**

**Additional file 4.**

## Data Availability

Upon completion of the RETAKE study, all data from the embedded process evaluation will be archived at the University of Nottingham. Any party may apply to the corresponding author for access to the data. Access will be governed by an information governance committee formed between The University of Nottingham and the University of Leeds.

## Abbreviations

FRESH: Facilitating Return to work through Early Specialist Health-based interventions
NHS: National Health Service
NPT: Normalisation Process Theory
OT: Occupational therapist
RA: Research Assistant
RCOT: Royal College of Occupational Therapists
RETAKE: RETurn to work After stroKE
SMS: Short Message Service
VR: Vocational Rehabilitation

## References

[1] Walker MF, Hoffmann TC, Brady MC, Dean CM, Eng JJ, Farrin AJ, Felix C, Forster A, Langhorne P, Lynch EA, Radford KA, Sunnerhagen KS, Watkins CL (2017). Improving the development, monitoring and reporting of stroke rehabilitation research: consensus-based core recommendations from the stroke recovery and rehabilitation roundtable. Int J Stroke.

[2] Dopp CME, Graff MJL, Teerenstra S, Adang EMM (2011). Nijhuis-van der Sanden MWG, Olde Rikkert MGM, et al. a new combined strategy to implement a community occupational therapy intervention: designing a cluster randomized controlled trial. BMC Geriatr.

[3] National Institute for Health Research (NIHR) (2015). Clinical trials guide for trainees: guidance to support NIHR trainees interested in getting involved in clinical trials.

[4] Holmes JA, Logan P, Morris R, Radford K (2020). Factors affecting the delivery of complex rehabilitation interventions in research with neurologically impaired adults: a systematic review. Syst Rev.

[5] Radford KA, Sutton C, Sach T, Holmes J, Watkins CL, Forshaw D, et al. Early, specialist vocational rehabilitation to facilitate return to work after traumatic brain injury: the FRESH feasibility RCT. Health Technol Assess. 2018;22(33). 10.3310/hta22330 PMC6004540 29863459

[6] Craig P, Dieppe P, Macintyre S, Michie S, Nazareth I, Petticrew M (2013). Developing and evaluating complex interventions: the new Medical Research Council guidance. Int J Nurs Stud.

[7] Holmes JA (2018). Implementing complex rehabilitation interventions in research: the example of vocational rehabilitation for people with traumatic brain injury. PhD [dissertation].

[8] Swindle T, Selig JP, Rutledge JM, Whiteside-Mansell L, Curran G (2018). Fidelity monitoring in complex interventions: a case study of the WISE intervention. Arch Public Health.

[9] LaRocca R, Yost J, Dobbins M, Ciliska D, Butt M (2012). The effectiveness of knowledge translation strategies used in public health: a systematic review. BMC Public Health.

[10] Lal S, Korner-Bitensky N (2012). Motivational interviewing: a novel intervention for translating rehabilitation research into practice. Disabil Rehabil.

[11] Damschroder LJ, Aron DC, Keith RE, Kirsh SR, Alexander JA, Lowery JC (2009). Fostering implementation of health services research findings into practice: a consolidated framework for advancing implementation science. Implement Sci.

[12] Jones CA, Roop SC, Pohar SL, Albrecht L, Scott SD (2015). Translating knowledge in rehabilitation: systematic review. Phys Ther.

[13] Farmer AP, Légaré F, Turcot L, Grimshaw J, Harvey E, McGowan JL (2008). Printed educational materials: effects on professional practice and health care outcomes. Cochrane Database Syst Rev.

[14] Grimshaw J, Eccles M, Thomas R, MacLennan G, Ramsay C, Fraser C, Vale L (2006). Toward evidence-based quality improvement. Evidence (and its limitations) of the effectiveness of guideline dissemination and implementation strategies 1966-1998. J Gen Intern Med.

[15] Effective Practice and Organisation of Care (EPOC). EPOC Taxonomy. https://epoc.cochrane.org/epoc-taxonomy. Accessed 15^th^ March 2021.

[16] Effective Practice and Organisation of Care (EPOC). *The EPOC taxonomy of health systems interventions. EPOC Resources for review authors*. https://epoc.cochrane.org/sites/epoc.cochrane.org/files/public/uploads/taxonomy/epoc_taxonomy_guidance.pdf. Accessed 15th March 2021.

[17] Cahill LS, Carey LM, Lannin NA, Turville M, Neilson CL, Lynch EA (2020). Implementation interventions to promote the uptake of evidence-based practices in stroke rehabilitation. Cochrane Database Syst Rev.

[18] Flodgren G, O'Brien MA, Parmelli E, Grimshaw JM. Local opinion leaders: effects on professional practice and healthcare outcomes. Cochrane Database Syst Rev. 2019(6). Art. No.: CD000125. DOI: 10.1002/14651858.CD000125.pub5. Accessed 03 March 2021. 10.1002/14651858.CD000125.pub5 PMC6589938 31232458

[19] Holmes J, Fletcher-Smith J, Merchán-Baeza J, Phillips J, Radford K (2021). Can a complex vocational rehabilitation intervention be delivered with fidelity? Fidelity assessment in the FRESH feasibility trial. Pilot and Feasibility Studies.

[20] Radford KA, Craven K, McLellan V, Sach TH, Brindle R, Holloway I, Hartley S, Bowen A, O'Connor R, Stevens J, Philips J, Walker M, Holmes J, McKevitt C, Murray J, Watkins C, Powers K, Shone A, Farrin A (2020). An individually randomised controlled multi-Centre pragmatic trial with embedded economic and process evaluations of early vocational rehabilitation compared with usual care for stroke survivors: study protocol for the RETurn to work after stroKE (RETAKE) trial. Trials..

[21] National Institute for Health and Care Excellence (NICE) (2013). Stroke rehabilitation in adults: Clinical guideline [CG162].

[22] NHS England. The NHS long term plan. 2019. https://www.longtermplan.nhs.uk/wp-content/uploads/2019/08/nhs-long-term-plan-version-1.2.pdf. Accessed 15 March 2021.

[23] Stroke Association. NHS England long term plan and the National Stroke Programme n.d. https://www.stroke.org.uk/get-involved/campaigning/nhs-long-term-plan. Accessed 15 March 2021.

[24] Department of Health. National Stroke Strategy. 2007. https://www.england.nhs.uk/south/wp-content/uploads/sites/6/2017/07/national-stroke-strategy-2007.pdf. Accessed 15 March 2021.

[25] NHS Digital. NHS Outcomes Framework 2019/20 Indicator and Domain Summary Tables: NHS Digital 2020. https://files.digital.nhs.uk/43/6B7A4F/nhs-out-fram-feb-20-dash.pdf. .

[26] Grant M (2016). Developing, delivering and evaluating stroke specific vocational rehabilitation: a feasibility randomised controlled trial. PhD [dissertation].

[27] O’Brien BC, Harris IB, Beckman TJ, Reed DA, Cook DA (2014). Standards for reporting qualitative Research: a synthesis of recommendations. Acad Med.

[28] NHS Health Research Authority (2017). UK policy framework for Health and social care Research.

[29] World Medical Association (WMA) (1996). Declaration of Helsinki: WMA.

[30] Sinclair E, Radford KA, Grant M, Terry J (2014). What is a return to work after stroke? 12-month work outcomes in a feasibility trial. J Rehabil Med.

[31] May C, Rapley T, Mair FS, Treweek S, Murray E, Ballini L (2015). Normalization Process Theory On-line User's Manual, Toolkit and NoMAD instrument.

[32] May CR, Cummings A, Girling M, Bracher M, Mair FS, May CM, Murray E, Myall M, Rapley T, Finch T (2018). Using normalization process theory in feasibility studies and process evaluations of complex healthcare interventions: a systematic review. Implement Sci.

[33] Braun V, Clarke V (2006). Using thematic analysis in psychology. Qual Res Psychol.

[34] Ltd QIP (2018). NVivo qualitative data analysis software (version 12).

[35] Shenton A (2004). Strategies for ensuring trustworthiness in qualitative Research projects. Educ Inf.

[36] Bassey M (1981). Pedagogic research: on the relative merits of search for generalisation and study of single events. Oxf Rev Educ.

[37] Doyle NW, Gafni Lachter L, Jacobs K (2019). Scoping review of mentoring research in the occupational therapy literature, 2002–2018. Aust Occup Ther J.

[38] McCormack B, Rycroft-Malone J, Decorby K, Hutchinson AM, Bucknall T, Kent B (2013). A realist review of interventions and strategies to promote evidence-informed healthcare: a focus on change agency. Implement Sci.

[39] Sampson EL, Feast A, Blighe A, Froggatt K, Hunter R, Marston L (2020). Pilot cluster randomised trial of an evidence-based intervention to reduce avoidable hospital admissions in nursing home residents (Better Health in Residents of Care Homes with Nursing—BHiRCH-NH Study). BMJ Open.

[40] Department of Health and Social Care, The Executive Office, The Scottish Government, & Welsh Government. Saving and improving lives: The future of UK clinical research delivery. 2021. https://assets.publishing.service.gov.uk/government/uploads/system/uploads/attachment_data/file/971971/The-future-of-UK-clinical-research-delivery-final.pdf. Accessed 29 March 2021.

[41] Messner DA, Moloney R, Warriner AH, Wright NC, Foster PJ, Saag KG (2016). Understanding practice-based research participation: the differing motivations of engaged vs. non-engaged clinicians in pragmatic clinical trials. Contemporary Clin Trials Communications.

[42] Al-Tannir MA, Katan HM, Al-Badr AH, Al-Tannir MM, Abu-Shaheen AK (2018). Knowledge, attitudes, practices and perceptions of clinicians towards conducting clinical trials in an academic tertiary care center. Saudi Med J.

[43] Krishna R, Maithhreyi R, Surapaneni K (2010). Research Bias: a review for medical students. J Clin Diagn Res.

[44] Hooker L, Small R, Humphreys C, Hegarty K, Taft A (2015). Applying normalization process theory to understand implementation of a family violence screening and care model in maternal and child health nursing practice: a mixed method process evaluation of a randomised controlled trial. Implement Sci.

[45] Powell BJ, Fernandez ME, Williams NJ, Aarons GA, Beidas RS, Lewis CC, McHugh SM, Weiner BJ (2019). Enhancing the impact of implementation strategies in healthcare: a Research agenda. Front Public Health.

[46] Royal College of Occupational Therapists (RCOT). Health and Work Champions: Enabling staff to talk about work. 2020. file:///Users/kristellecraven/Downloads/Summary%20Health%20and%20Work%20Champions%20March%202020%20(1).pdf. Accessed 16 March 2021.

[47] Malterud K, Siersma VD, Guassora AD (2016). Sample size in qualitative interview studies: guided by information power. Qual Health Res.

