# Supplementary figures and images for: Embedding mentoring to support trial processes and implementation fidelity in a randomised controlled trial of vocational rehabilitation for stroke survivors
Source: BMC Med Res Methodol. 2021 Oct 3;21:203. doi: 10.1186/s12874-021-01382-y (PMC8487447; doi:10.1186/s12874-021-01382-y)

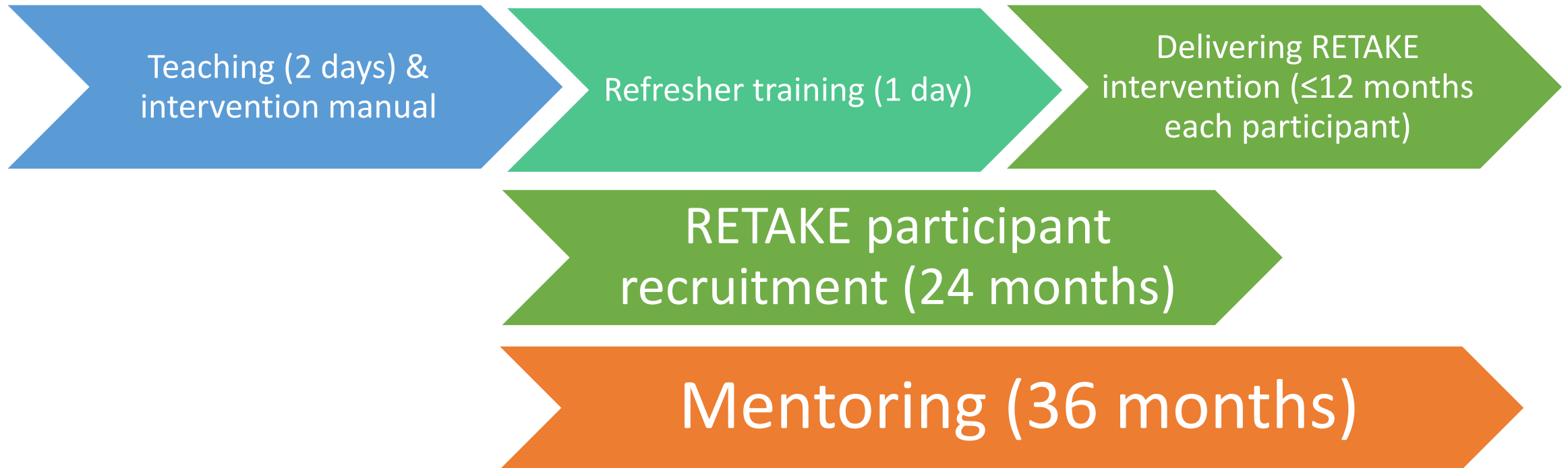

Additional file 1. Core elements of the RETAKE mentoring approach

Supplement: Supplementary file 1 — Additional file 1. [file 12874_2021_1382_MOESM1_ESM.pdf]
