# Supplementary material for: Embedding mentoring to support trial processes and implementation fidelity in a randomised controlled trial of vocational rehabilitation for stroke survivors
Source: BMC Med Res Methodol. 2021 Oct 3;21:203. doi: 10.1186/s12874-021-01382-y (PMC8487447; doi:10.1186/s12874-021-01382-y)
Supplement: Supplementary file 2 — Additional file 2. [file 12874_2021_1382_MOESM2_ESM.pdf]

RETAKE

Return to Work After Stroke

FORM 53 Therapist Mentor Record

To be completed by the ESSVR mentor following each mentor session

Mentor name

Date of session

Duration of session minutes

Method of session delivery

☐ Telephone / teleconference ☐ Face-to-face

☐ Other, please specify

Please specify who **was present** at the session:

**Full name** *(Please print)*	**Site name**	Office use only<br>**Therapist ID**

Please specify who **did not attend** the session:

**Full name** *(Please print)*	**Reason**	Office use only<br>**Therapist ID**

Clinical Issues and Actions

Prior to returning this form to CTRU you must make a copy of the form and any amendments for retention at site. CTRU, University of Leeds (please see Investigator Site File for full contact details).

Form continues on next page ►►

For office use only	Computerised		Verified/Checked	
	Date	Initials	Date	Initials

FORM 53 Therapist Mentor Record

Implementing ESSVR and Actions

Trial-related Issues and Actions

Prior to returning this form to CTRU you must make a copy of the form and any amendments for retention at site. CTRU, University of Leeds (please see Investigator Site File for full contact details).

Form continues on next page ►►

**For office use only**	Computerised		Verified/Checked	
	Date	Initials	Date	Initials

RETAKE Therapist Issues

Including any staffing issues e.g. extended periods of leave, change of therapist, identification of training needs

Any other Issues or Comments

Prior to returning this form to CTRU you must make a copy of the form and any amendments for retention at site. CTRU, University of Leeds (please see Investigator Site File for full contact details).

Form continues on next page ►►

For office use only	Computerised		Verified/Checked	
	Date	Initials	Date	Initials

Therapist Mentor Record

Any other Issues or Comments *(Continued)*

Completed by (Mentor)		Date	

Prior to returning this form to CTRU you must make a copy of the form and any amendments for retention at site. CTRU, University of Leeds (please see Investigator Site File for full contact details).

Last Page ■

For office use only	*Computerised*		*Verified/Checked*	
	Date	*Initials*	*Date*	*Initials*
