# Supplementary material for: Embedding mentoring to support trial processes and implementation fidelity in a randomised controlled trial of vocational rehabilitation for stroke survivors
Source: BMC Med Res Methodol. 2021 Oct 3;21:203. doi: 10.1186/s12874-021-01382-y (PMC8487447; doi:10.1186/s12874-021-01382-y)
Supplement: Supplementary file 3 — Additional file 3. [file 12874_2021_1382_MOESM3_ESM.pdf]

Topic Guide for Semi-Structured interviews with RETAKE Mentors

Intro: Thank you for participating in the study and for giving up this time, we value your contribution. Today I am interested in finding out your experience of being a mentor for the RETAKE OT's. Please feel free to discuss anything you think is relevant.

Background/previous experience

1. Can you start by telling me about your background in OT? *(Collective action: skill set workability)*
    a. What previous experience do you have with VR?
    b. Do you have any previous experience in mentoring roles?
        i. *If so, did this help you in your RETAKE role?*
        ii. *If not, do you think this made things difficult?*
    c. What is your previous experience of research before taking part in RETAKE?
2. Did you feel equipped for this role? *(Collective action: skill set workability) – or legitimation?*
    i. *Did you feel you had the right amount of knowledge skills/experience?*
    ii. *If not - what might have better prepared you?*
3. Did you receive any support from the RETAKE study team to help with the mentoring?
    i. *If not, what support might have helped?*

Experiences of the mentoring process

4. How would you describe your role as a mentor? (*Coherence: individual specification)*
    a. In addition to the scheduled mentoring sessions, how much extra support have the RETAKE OTs required?
    b. What topics or questions tend to come up in mentoring sessions?
        i. *Did they need any support to understand the aims and potential benefits of the intervention? (Coherance: communal specification)*
        ii. *Did the OT's question the differences between ESSVR (the trial intervention) and their usual care roles? (Coherence: Differentiation*
    c. How did the OTs report coping with balancing the RETAKE work and their usual workload? *(Collective action: contextual integration)*
    d. Do you think the OTs' had the right skills and experience to be delivering this kind of intervention? *(Collective action: skill set workability)*
5. Do you think the mentoring is helpful to the RETAKE OTs?
    a. If so, in what ways is this helpful?
        i. ***[if they answer no]…*** *Why not?*

ii. ***[if they answer yes]…***
If you could single out the active ingredients of mentoring, what would you say they are? (note: an active ingredient is something that leads to the intervention having an effect on the desired outcome – e.g., getting the OTs to deliver the intervention as intended)

b. What do you think could have made it more helpful?

<u>Barriers and facilitators to delivery and implementation</u>

6. How have you found balancing the mentoring duties with your existing workload? *(Collective action: contextual integration) (Reflexive monitoring: individual appraisal)*
7. What barriers do you come across while delivering mentoring?
    a. Did RETAKE OTs engage with the mentoring process?
        i. If not, why do you think this was?
    b. What reasons did OT's give for not attending mentoring sessions?
8. The mentoring was in the form of group sessions and phone calls, what did you think about this format?
9. From your understanding of the RETAKE intervention, are there any similar therapies available in your service or otherwise in your area? *(Coherence: Differentiation)*
10. If the ESSVR intervention was set up within your service, and mentoring was considered a critical aspect of its success, how do you think this would be received and accommodated? *(Collective action: interactional workability)*
    a. What changes would need to be made to the process to make it roll out more smoothly?
11. Have you gained anything through being a mentor? *(Reflexive monitoring: individual appraisal)*
12. Do you feel your participation was a worthwhile use of your time in this case? *(Reflexive monitoring: individual appraisal)*
