# Supplementary material for: Embedding mentoring to support trial processes and implementation fidelity in a randomised controlled trial of vocational rehabilitation for stroke survivors
Source: BMC Med Res Methodol. 2021 Oct 3;21:203. doi: 10.1186/s12874-021-01382-y (PMC8487447; doi:10.1186/s12874-021-01382-y)
Supplement: Supplementary file 4 — Additional file 4. [file 12874_2021_1382_MOESM4_ESM.pdf]

RETAKE - RETurn to work After stroKE

THERAPIST TOPIC GUIDE – INTERVIEWS

Opening/introduction:

Thanks for participating in the study and for giving up this time.

Reminder of the focus of study

---

1. Can you describe the aim of the intervention?

2. Can you describe the mechanism of the intervention – how was it supposed to work?

3. Did this make it different to usual care, and if so how?

4. Was there any part of the intervention that you consider was unnecessary? If yes, why?

5. Was there any further content to the intervention that you think should be included? If yes, why?

---

6. Do you think this intervention was useful?

7. Did you find the training (manual, teaching, mentoring) useful; and did it equip you to deliver the intervention?

8. Was taking part in the training a worthwhile investment of your time?

---

9. How compatible is the intervention with the existing stroke care pathway?

10. In relation to any other local RTW services, did this intervention complement those services, duplicate them or fill a gap?

11. Can you describe how managers, colleagues and external organisations (including employer) supported you?

---

12. Can you tell me about your experience of delivering this intervention overall?
    - time/resources invested in delivery vs impact
    - barriers to delivering the intervention; and how to address barriers?

14. Were you able to see that there were any benefits or dis-benefits, and what were these?

15. Did you learn anything about what would be needed to roll out the intervention effectively?
    - changes to intervention; changes in services/resources needed for delivery)
